# Psychotic Like Experiences in Healthy Adolescents are Underpinned by Lower Fronto-Temporal Cortical Gyrification: a Study from the IMAGEN Consortium

**DOI:** 10.1093/schbul/sbac132

**Published:** 2022-10-05

**Authors:** Raka Maitra, Charlotte M Horne, Owen O’Daly, Evangelos Papanastasiou, Christian Gaser, IMAGEN list of authors, Tobias Banaschewski, Gareth J Barker, Arun L W Bokde, Sylvane Desrivières, Herta Flor, Antoine Grigis, Hugh Garavan, Penny Gowland, Andreas Heinz, Rüdiger Brühl, Jean-Luc Martinot, Marie-Laure Paillère Martinot, Eric Artiges, Frauke Nees, Dimitri Papadopoulos Orfanos, Herve Lemaitre, Tomáš Paus, Luise Poustka, Sarah Hohmann, Sabina Millenet, Juliane H Fröhner, Lauren Robinson, Michael N Smolka, Henrik Walter, Jeanne M Winterer, Robert Whelan, Erin Burke Quinlan, Alex Ing, Gunter Schumann, IMAGEN Consortium, Sukhi Shergill

**Affiliations:** Department of Psychosis Studies, Institute of Psychiatry, Psychology and Neuroscience, King’s College London, UK; Department of Psychosis Studies, Institute of Psychiatry, Psychology and Neuroscience, King’s College London, UK; Department of Neuroimaging, Institute of Psychiatry, Psychology and Neuroscience, King’s College London, UK; Department of Psychosis Studies, Institute of Psychiatry, Psychology and Neuroscience, King’s College London, UK; Therapeutic Area CNS, Boehringer Ingelheim International GmbH, Inghelheim, Germany; Departments of Neurology, Jena University Hospital, Jena, Germany; Departments of Psychiatry and Psychotherapy, Jena University Hospital, Jena, Germany; Department of Child and Adolescent Psychiatry and Psychotherapy, Central Institute of Mental Health, Medical Faculty Mannheim, Heidelberg University, Mannheim, Germany; Department of Neuroimaging, Institute of Psychiatry, Psychology and Neuroscience, King’s CollegeLondon, UK; Discipline of Psychiatry, School of Medicine and Trinity College Institute of Neuroscience, Trinity College Dublin, Dublin, Ireland; Centre for Population Neuroscience and Precision Medicine (PONS), Institute of Psychiatry, Psychology and Neuroscience, Social, Genetic and Developmental Psychiatry Centre, King’s CollegeLondon, UK; Institute of Cognitive and Clinical Neuroscience, Central Institute of Mental Health, Medical Faculty Mannheim, Heidelberg University, Mannheim, Germany; Department of Psychology, School of Social Sciences, University of Mannheim, Mannheim, Germany; NeuroSpin, CEA, Université Paris-Saclay, Gif-sur-Yvette, France; Departments of Psychiatry and Psychology, University of Vermont, Burlington, Vermont, USA; Sir Peter Mansfield Imaging Centre School of Physics and Astronomy, University of Nottingham, University Park, Nottingham, UK; Department of Psychiatry and Psychotherapy CCM, Charité—Universitätsmedizin Berlin, Freie Universität Berlin, Humboldt-Universität zu Berlin, and Berlin Institute of Health, Berlin, Germany; Physikalisch-Technische Bundesanstalt (PTB), Braunschweig and Berlin, Germany; Institut National de la Santé et de la Recherche Médicale, INSERM U 1299 “Trajectoires développementales en psychiatrie”; Université Paris-Saclay, Ecole Normale supérieure Paris-Saclay, CNRS, Centre Borelli, Gif-sur-Yvette, France; Institut National de la Santé et de la Recherce Médicale, INSERM U 1299 “Trajectoires développementales and psychiatrie”, University Paris-Saclay, Ecole Normale Supérieure Paris-Saclay, CNRS; Centre Borelli, Gif-sur-Yvette, France; and AP-HP. Sorbonne Université, Department of Child and Adolescent Psychiatry, Pitié-Salpêtrière Hospital, Paris, France; Institut National de la Santé et de la Recherche Médicale, INSERM U 1299 “Trajectoires développementales en psychiatrie”; Université Paris-Saclay, Ecole Normale supérieure Paris-Saclay, CNRS, Centre Borelli, Gif-sur-Yvette; and Psychiatry Department, EPS Barthélémy Durand, Etampes, France; Department of Child and Adolescent Psychiatry and Psychotherapy, Central Institute of Mental Health, Medical Faculty Mannheim, Heidelberg University, Mannheim, Germany; Institute of Cognitive and Clinical Neuroscience, Central Institute of Mental Health, Medical Faculty Mannheim, Heidelberg University, Mannheim, Germany; PONS Research Group, Dept of Psychiatry and Psychotherapy, Campus Charite Mitte, Humboldt University, Berlin, Germany; NeuroSpin, CEA, Université Paris-Saclay, Gif-sur-Yvette, France; NeuroSpin, CEA, Université Paris-Saclay, Gif-sur-Yvette, France; Institut des Maladies Neurodégénératives, UMR 5293, CNRS, CEA, Université de Bordeaux, Bordeaux, France; Departments of Psychiatry and Neuroscience, Faculty of Medicine and Centre Hospitalier Universitaire Sainte-Justine, University of Montreal, Montreal, Quebec, Canada; Departments of Psychology and Psychiatry, University of Toronto, Toronto, Ontario, Canada; Department of Child and Adolescent Psychiatry and Psychotherapy, University Medical Centre Göttingen, Göttingen, Germany; Department of Child and Adolescent Psychiatry and Psychotherapy, Central Institute of Mental Health, Medical Faculty Mannheim, Heidelberg University, Mannheim, Germany; Department of Child and Adolescent Psychiatry and Psychotherapy, Central Institute of Mental Health, Medical Faculty Mannheim, Heidelberg University, Mannheim, Germany; Department of Psychiatry and Neuroimaging Center, Technische Universität Dresden, Dresden, Germany; Department of Psychological Medicine, Section for Eating Disorders, Institute of Psychiatry, Psychology and Neuroscience, King’s College London, London, UK; Department of Psychiatry and Neuroimaging Center, Technische Universität Dresden, Dresden, Germany; Department of Psychiatry and Psychotherapy CCM, Charité—Universitätsmedizin Berlin, Freie Universität Berlin, Humboldt-Universität zu Berlin, and Berlin Institute of Health, Berlin, Germany; Department of Psychiatry and Psychotherapy CCM, Charité—Universitätsmedizin Berlin, Freie Universität Berlin, Humboldt-Universität zu Berlin, and Berlin Institute of Health, Berlin, Germany; Department of Education and Psychology, Freie Universität Berlin, Berlin, Germany; School of Psychology and Global Brain Health Institute, Trinity College Dublin, Dublin, Ireland; Centre for Population Neuroscience and Precision Medicine (PONS), Institute of Psychiatry, Psychology and Neuroscience, Social, Genetic and Developmental Psychiatry Centre, King’s CollegeLondon, UK; Centre for Population Neuroscience and Precision Medicine (PONS), Institute of Psychiatry, Psychology and Neuroscience, Social, Genetic and Developmental Psychiatry Centre, King’s CollegeLondon, UK; Centre for Population Neuroscience and Precision Medicine (PONS), Institute of Psychiatry, Psychology and Neuroscience, Social, Genetic and Developmental Psychiatry Centre, King’s CollegeLondon, UK; PONS Research Group, Dept of Psychiatry and Psychotherapy, Campus Charite Mitte, Humboldt University, Berlin, Germany; Leibniz Institute for Neurobiology, Magdeburg, Germany; Institute for Science and Technology of Brain-inspired Intelligence (ISTBI), Fudan University, Shanghai, P.R. China; Department of Psychosis Studies, Institute of Psychiatry, Psychology and Neuroscience, King’s CollegeLondon, UK; Kent and Medway Medical School, Canterbury, UK

**Keywords:** cortical gyrification, adolescence, psychotic like experiences, IMAGEN, MRI, psychosis

## Abstract

**Background and Hypothesis:**

Psychotic Like Experiences (PLEs) are widely prevalent in children and adolescents and increase the risk of developing psychosis. Cortical gyrification characterizes brain development from in utero till about the first 2 years of life and can be measured in later years as *static gyrification* changes demonstrating neurodevelopment and *dynamic gyrification* changes reflecting brain maturation during adolescence. We hypothesized that PLEs would be associated with *static cortical gyrification* changes reflecting a neurodevelopmental abnormality.

**Study Design:**

We studied 1252 adolescents recruited in the IMAGEN consortium. We used a longitudinal study design, with Magnetic Resonance Imaging measurements at age 14 years and age 19 years; measurement of PLEs using the Community Assessment of Psychic Experiences (CAPE) questionnaire at age 19 years; and clinical diagnoses at age 23 years.

**Study Results:**

Our results show *static gyrification* changes in adolescents with elevated PLEs on 3 items of the CAPE—voice hearing, unusual experiences of receiving messages, and persecutory ideas—with lower cortical gyrification in fronto-temporal regions in the left hemisphere. This group also demonstrated *dynamic gyrification* changes with higher cortical gyrification in right parietal cortex in late adolescence; a finding that we replicated in an independent sample of patients with first-episode psychosis. Adolescents with high PLEs were also 5.6 times more likely to transition to psychosis in adulthood by age 23 years.

**Conclusions:**

This is the largest study in adolescents that demonstrates fronto-temporal abnormality of cortical gyrification as a potential biomarker for vulnerability to PLEs and transition to psychosis.

## Introduction

Psychotic symptoms exist on a continuum, from Psychotic Like Experiences (PLE) at one end of the spectrum to clinical phenotypes such as schizophrenia at the other end. As the definition of PLEs varies,^1^ here we refer to PLEs as subtle, subclinical symptoms of psychosis (ie, in otherwise healthy, nontreatment seeking-individuals) that are present in the general population including perceptual disturbances, paranoid ideation, and “magical” thinking.^2^ PLEs offer a compelling model for subclinical psychosis because they are phenomenologically similar to psychotic illness, are heritable, share common genetic influences with depression and schizophrenia,^3^ and overlap with other risk factors for schizophrenia such as childhood trauma, victimization, and substance use.^4^ If PLEs arise from abnormalities of brain structure that predispose to dysfunction within cognitive and perceptual networks, then detecting such structural abnormalities may help understand the mechanisms underpinning psychosis and identify individuals at risk of developing psychosis.

PLEs are common in childhood, occurring in 17% of children aged 9–12 years,^5^ and have their highest prevalence in younger adolescents (21%–23%) compared to older adolescents (7%).^6^ Presence of PLEs in children aged 11 years may be a significant predictor of schizophreniform disorder at age 26 years.^7^ A large study of 3801 young adults reported that experience of hallucinations at age 14 years, especially in the presence of childhood behavior problems, was a predictor of delusional experiences and non-affective psychosis at age 21 years.^8,9^ Overall, adolescents with PLEs were 3.5 times more likely to transition to a clinical psychotic disorder in adulthood compared to adolescents with no PLEs^10^ and the association between PLEs and a diagnosable psychiatric disorder was shown to increase with age—where 57% of early adolescents (aged 11–13 years) and 80% of mid adolescents (aged 13–16 years) with reported psychotic symptoms had at least one diagnosis.^6^

Adolescence is marked by significant neurodevelopmental changes that may be reflected in structural changes in the brain. Structural brain abnormalities have been noted in adolescents experiencing PLEs^11,12^ with some of these structural changes being associated with genetic loading.^3,13^ Cortical gyrification is a developmental process that refers to the folding of the cortex into gyri and sulci. Gyrification increases rapidly in-utero until 2 years after birth and then remains relatively constant therefore offering a stable marker of neurodevelopmental changes.^14^ We refer to this stable type of gyrification as a result of early neurodevelopmental changes as “static” gyrification. There also may be further changes in cortical gyrification that occur later such as nonlinear decreases in gyrification^15^ although the processes that contribute to these later changes in gyrification are less well understood. Here we refer to this later maturational aspect of gyrification as “dynamic” gyrification. Abnormalities of gyrification may be associated with persistence of PLEs in adolescents and increased risk of developing psychosis.^16^

Only a handful of studies, mostly in young adults, have explored gyrification as a neurodevelopmental marker for risk of developing psychosis (see Appendix in Supplement). While the gyrification data presents a mixed picture, the main regional findings are alterations reported in the frontal, temporal, and parietal lobes, with some decreases in gyrification suggesting that an early neurodevelopmental change results in the persistence of psychotic symptoms^16–18^; and other studies reporting higher gyrification^19,20^ as well as no changes in gyrification.^21,22^ A recent review of these gyrification findings in ultra-high risk and early psychosis populations report higher gyrification in frontal, temporal, and parietal regions while there may be more significant decreases evident in the established disease.^23^ Another study suggests higher gyrification in early psychosis with less clear findings in those at high risk for psychosis.^24^ In these cross-sectional studies, the differentiation between static and dynamic gyrification changes and their implication in psychosis remain unclear.

Our aim was to study abnormalities in static and dynamic cortical gyrification in healthy adolescents experiencing PLEs compared to those not experiencing PLEs. We measured cortical gyrification from structural magnetic resonance imaging (MRI) scans collected at baseline (14 years) and follow-up (19 years) from 1252 adolescents that participated in the IMAGEN consortium.^25^ PLEs were assessed at follow-up (19 years) using the Community Assessment of Psychic Experiences (CAPE-42).^26^ We also investigated whether differences in cortical gyrification associated with PLEs were relevant to clinically diagnosed psychotic symptoms by comparing cortical gyrification in an independent sample of patients with first-episode psychosis and healthy controls (HC), and assessing how many high PLE adolescents transitioned to psychosis illness at a final follow-up phase (age 23 years).

We hypothesized that:

1) Adolescents with PLEs, with high CAPE scores, would show a neurodevelopmental abnormality as indexed by altered frontal and temporoparietal static cortical gyrification at baseline compared to those without PLEs.
2) Adolescents with PLEs, with high CAPE scores, would show a neurodevelopmental abnormality as evidenced by persistently altered frontal and temporoparietal static cortical gyrification from baseline to follow-up compared to adolescents without PLEs.
3) Altered static cortical gyrification in adolescents with PLEs would show clinical relevance to symptoms of psychosis as indexed by a) similarly altered static cortical gyrification in an independent sample of patients with early psychosis compared to HC, and b) a higher rate of transition to psychosis at follow-up in the adolescents experiencing elevated levels of PLEs.^10^

## Methods

### Participants

A total of 1252 adolescents were selected from the IMAGEN consortium^25^ based on availability of a complete behavioral and neuroimaging data set at ages 14 (baseline) and 19 years (follow-up) and additional behavioral data at age 23 years. Ethical approval had been obtained by the ethics research committee of the IMAGEN consortium.^25^ Access to the IMAGEN database was obtained after approval by the IMAGEN committee and deidentified data were downloaded. The present study included participants that were recruited between January 1, 2016 and January 1, 2017.

### Sample Stratification

Participants were stratified into “high” and “low” PLE groups on the basis of scores of selected items from the CAPE questionnaire as described in our previous publication.^26^ The CAPE questionnaire is a 42-item questionnaire that has been shown to have good validity and reliability for measuring PLEs as reported in a recent meta-analysis of 111 studies.^27,28^ Briefly, two complementary stratifications were performed. First, high and low scorers were determined based on the upper and lower quartiles of the composite scores of items-5 (bizarre experiences), 7 (perceptual abnormalities), and 33 (persecutory ideas) from the CAPE questionnaire (hereon referred to as the “CAPE-3” sample). This stratification was based on 2 studies showing these 3 factors are most important for predicting poor outcomes in adolescents experiencing PLEs^28,29^ (see Supplementary Materials). Second, the upper and lower deciles of the total CAPE scores (hereon referred to as the “CAPE-42” sample) were used to determine high and low PLE scorers.

### Measures of IQ and General Psychopathology

IQ was measured using the Wechsler Intelligence Scale for Children (WISC, IV),^30^ alcohol consumption using the Alcohol Use Disorders Identification Test (AUDIT),^31,32^ cannabis use using the Drug Abuse Screening Test (DAST),^33^ and depressive symptoms using the Adolescent Depression Rating Scale (ADRS).^34^ WISC scores were available only at baseline while CAPE, ADRS, AUDIT, and DAST scores were available only at follow-up.

### MRI Acquisition

MRI data were acquired from the following participating centers for IMAGEN study: Institute of Psychiatry, University of Nottingham (UK); Charité University Berlin, Technical University Dresden, University Medical Centre Hamburg-Eppendorf, Central Institute of Mental Health Mannheim (Germany); National Institute of Health and medical Research (France); and Trinity College Dublin (Ireland) using 3T scanners from four different manufacturers namely Siemens, Philips, GE Healthcare and Bruker. A full description of the scanning protocols, cross-site standardization and quality checks, and pre-processing of resulting data are provided elsewhere.^35^

### Cortical Gyrification

The cortical surface was extracted and local gyrification was measured across the whole brain using the CAT12 toolbox (http://dbm.neuro.uni-jena.de/cat/) in SPM12 software (http://www.fil.ion.ucl.ac.uk/spm, Wellcome Trust Centre for Neuroimaging), (see Supplementary Materials). This method for calculating a local gyrification index is based on the absolute mean curvature of the brain surface and was chosen as a simple measure that is sensitive to both amplitude and frequency of the folding pattern.^36^ CAT12 has also previously been used to explore gyrification in adults with PLEs.^37^ To ensure normal distribution of gyrification indices, smoothing was applied using a Gaussian kernel of 20 mm FWHM (the recommended CAT12 filter size) prior to statistical analyses. The results are displayed using the Desikan atlas.^38^

### Statistical Analysis

Demographics, measures of IQ, and general psychopathology were analyzed using SPSS.^39^

All statistical analyses of neuroimaging data were performed using SPM12 (http://www.fil.ion.ucl.ac.uk/spm; Wellcome Trust Centre for Neuroimaging). A flexible factorial model was used with CAPE score grouping (ie CAPE-3 or CAPE-42) as the between-subjects factor (high and low scorers) and TIME as the within-subjects factor (baseline and follow-up visits) to explore the main effect of group, time, and the interaction between group and time.

Neuroimaging results were considered at a statistical cluster height threshold of *P* < .001 uncorrected, with the cluster extent of 50 vertices—similar to previous gyrification studies.^40,41^ Main effects reported at an uncorrected threshold were then subjected to more statistically stringent post-hoc tests using non-parametric methods such as Threshold Free Cluster Enhancement (TFCE) using a threshold of *P* < .001 and 5000 permutations.^42^

### Independent Replication Sample

To examine whether the mechanism underlying PLEs was the same as that in clinical psychosis, we performed a cross-sectional replication analysis in an independent sample of 102 medicated patients with early psychosis (duration of illness <5 years) compared to 43 HC collected as part of the MUTRIPS study.^43^

### Follow-up Diagnoses

Finally, we assessed the utility of the high and low CAPE-3 classification by examining how many adolescents in each group developed psychosis at a clinical follow-up at age 23 years. Clinical diagnoses were established using the Mini-International Neuropsychiatric Interview (M.I.N.I.).^44^

## Results

### Demographics

Demographics for each sample stratification are available in Table 1. Briefly, for the CAPE-3 sample stratification there was a total sample of 600 adolescents with 313 high CAPE-3 scorers and 287 low CAPE-3 scorers. There were no significant differences in age, IQ (WISC IV), alcohol use (AUDIT), and cannabis use (DAST) scores between the high and low CAPE-3 scorers. Only the ADRS scores differed between the 2 groups (*Z* = 9.7, *P* < .001) where low CAPE-3 scorers displayed higher depressive symptoms than high CAPE-3 scorers.

**Table 1.** Demographics of the Participants

	CAPE-3		Comparison Between CAPE-3 High and Low Scorers	CAPE-42		Comparison Between CAPE-42 High and Low Scorers	Entire Sample	Correlation of CAPE Scores With Other Measures in the Entire Sample
No of participants (*n*)	600			247			1252	
Stratification	High CAPE-3	Low CAPE-3		High CAPE-42	Low CAPE-42		–	
Stratification (*n*)	313	287		123	124		1252	
Age (mean, SD) BL	14.4, 0.4	14.4, 0.4		14.4, 0.4	14.4, 0.4		14.4, 0.4	
Age (mean, SD) FU	18.9, 0.7	18.9, 0.7		18.9, 0.7	18.9, 0.7		18.9, 0.7	
Gender (M, F, NK)	127, 180, 6	160, 123, 4		39, 84, 0	69, 55, 0		591, 651, 10	
Handedness (R, L, NK)	263, 39, 11	239, 37, 11		107, 13, 3	104, 18, 2		1090, 141, 21	
WISC Verbal (mean, SD) BL	111.2, 15.4	109.6, 15.1	n.s	112.2, 15.2	106.9, 15.2	*t* (*df* = 238) = 2.65, *P* = .008	112.5, 14.7	(*r* = 0.10, *P* < .001)
WISC performance (mean, SD) BL	108.8, 14.9	106.5, 15.3	n.s	109.5, 14.9	105.1, 14.6	*t* (*df* = 239) = 2.29, *P* = .023	108.9, 14.3	(*r* = 0.094, *P* = .001).
ADRS (mean, SD) FU	18.1, 2.5	19.7, 0.7	(*Z* = 9.7, *P* < .001)	16, 2.9	19.7, 0.6	(z= 11, p<0.001)	18.7, 2.1	(ρ= -0.492, p<0.001
AUDIT (mean, SD) FU	5.9, 4.4	5.5, 4.2	n.s	7.4, 5.2	5.2, 3.7	(*z* = −3.35, *P* = .001)	5.6, 4.1	(*ρ* = 0.079, *P* = .005)
DAST (mean, SD) FU	1.1, 2.2	0.6, 1.2	n.s	1.7, 2.7	0.6, 1.0	(*z* = −2.95, *P* = .003)	0.9, 1.8	(*ρ* = 0.097, *P* = .001)

*Note*: n.s., not significant; *Z*, standardized score of nonparametric comparison; *ρ*, spearman correlation coefficient; *r*, Pearson’s correlation coefficient; *n*, number; SD, standard deviation; BL, baseline; FU, follow-up; M, male; F, female; R, right; L, left; NK, not known; ADRS, Adolescent Depression Rating Scale; AUDIT, Alcohol Use Disorders Identification Test; CAPE-42, Community Assessment of Psychic Experiences Questionnaire, 42 items instrument; DAST, Drug Abuse Screening Test for Cannabis; WISC, Wechsler Intelligence Scale for Children.

### High Versus Low CAPE-3 Scorers

**Fig. 1.**
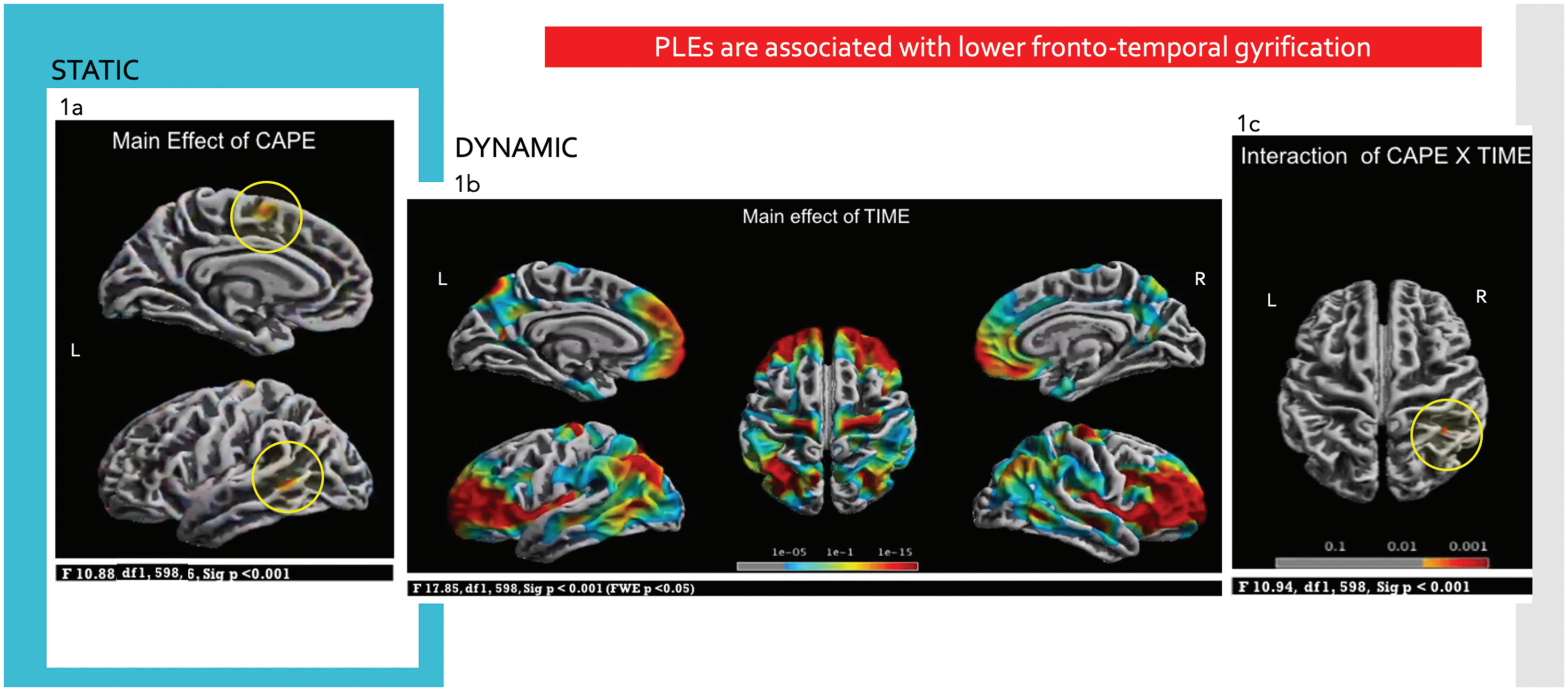
PLEs in adolescence are associated with lower fronto-temporal gyrification. (a) Differences in STATIC gyrification in fronto-temporal areas in high CAPE-3 scorers compared to low CAPE-3 scorers. (b) Changes in DYNAMIC gyrification from mid (baseline, 14 years) to late adolescence (follow-up, 19 years) reflecting maturational processes, most pronounced in prefrontal areas. (c) CAPE-3 × TIME interaction showing changes in DYNAMIC gyrification where high CAPE-3 scorers showed higher gyrification in parietal areas at follow-up. Results reported at *P* < .001.

Using a flexible factorial model, there was a main effect of CAPE on (static) cortical gyrification in the banks of the left superior frontal gyrus, precentral gyrus, superior temporal sulcus, and middle temporal gyrus (F(1,598) = 10.88, *P* < .001). Post-hoc *t*-tests showed that the high scorers on the CAPE-3 had lower gyrification in these areas compared to low scorers (t(1,598) = 3.10, *P* < .001) (figure 1a) where effects in the left precentral gyrus and left middle temporal gyrus were the most robust (*P* < .001 TFCE). Adding gender and scanning site as covariates to the model did not provide additional findings.

There was also a main effect of TIME (ie, across the whole CAPE-3 sample) where there were changes in (dynamic) cortical gyrification at follow-up, compared to baseline, in bilateral dorsal middle frontal, superior frontal, precentral, superior parietal, inferior parietal, precuneus, insula, and lingual gyrus; right postcentral, supramarginal, superior temporal, middle temporal, and entorhinal cortex. The observed changes in cortical gyrification were statistically significant (F(1,598) = 17.85, FWE corrected *P* < .05) and were most marked in prefrontal areas (figure 1b). Post hoc analysis (T(1,599) = 4.51, FWE corrected *P* < .05) confirmed increased gyrification at follow-up in the above areas and small areas of reduced gyrification in bilateral postcentral, superior parietal, and fusiform; and insula in right hemisphere.

CAPE × TIME interaction on (dynamic) cortical gyrification was seen in right superior and inferior parietal lobules (F(1,598) = 10.94, *P* < .001) (figure 1c). Post-hoc independent sample *t*-tests for baseline and follow-up visits separately showed that high scorers had higher gyrification in these regions compared to low scorers at follow-up (t(1,598) = 3.10, *P* < .001) but not at baseline. This finding also survived the more stringent statistical threshold at *P* < .001 TFCE.

We performed additional analysis with the entire sample of 1252 adolescents and with CAPE-42 10th decile stratification which did not offer additional relevant information (see supplement) and both of these samples had significant confounding variables (see Table 1).

### Independent Replication Sample

Demographics have been described in our previous article.^43^ The independent sample of patients with psychosis demonstrated higher gyrification in cuneus, caudal middle frontal in the left hemisphere and lingual/fusiform, lateral occipital/cuneus and superior parietal in the right hemisphere compared to HC (t(1,142) = 3.15, *P* < .001).

### Follow-up Diagnoses

There were 1252 adolescents included in this study, of whom 756 had follow-up data at age 23 years and of these, 16 adolescents received a diagnosis of psychosis at follow-up (2.1%). There were 12 (75%) from the high CAPE-3 group (totaling 187), and 2 (12.5%) from the low CAPE-3 group (totaling 177); and 2 (12.5%) from the remaining adolescents (intermediate CAPE-3 group). The high CAPE-3 scorers were 5.6 times more likely to develop psychosis than low CAPE-3 scorers (relative risk ratio = (12/187)/(2/177) = 5.68).^45^ In comparison, using the CAPE-42 classification there were 6 (37.5%) that transitioned from the high CAPE-42 group, 9 (56.25%) from intermediate group and 1 (6%) from low CAPE-42 group. This supports the increased value of using the more specific CAPE-3 item scores to identify adolescents at increased risk of developing psychosis.

## Discussion

Our longitudinal study presents a unique and powerful design exploring changes in gyrification during adolescence and differentiates between *static* and *dynamic gyrification* and their association with PLEs. We show that adolescents with high PLEs, especially with experiences of voice-hearing, receiving unusual messages and persecutory ideas (CAPE-3), have lower gyrification in fronto-temporal regions that persist through mid to late adolescence compared to adolescents with low PLEs thus reflecting *static gyrification* changes. Moreover, this high PLE group displayed higher gyrification in right superior and inferior parietal cortex at follow-up where cortical gyrification in these parietal regions was also altered (higher) in an independent sample of patients with early psychosis compared to controls, thus reflecting *dynamic gyrification* changes. The utility of using the more specific CAPE items (CAPE-3) to stratify participants was supported by the observation that 75% of the 16 adolescents that transitioned to clinical psychosis at age 23 years were in the high PLE group (relative risk ratio of 5.6). Together, this suggests that the neurodevelopmental abnormalities associated with high PLEs are clinically relevant to symptoms of psychosis.

Few studies have explored gyrification as a neurodevelopmental marker for risk of developing psychosis but indicate that an early neurodevelopmental insult results in an earlier plateau of cortical expansion which underlies persistence of psychotic symptoms.^16–18^ However, other studies have reported both increases^19,20^ and no changes in gyrification.^21,22^ Recently, Fonville et al reported that young adults with persistent PLEs (at age 18 and 20 years) showed lower gyrification in left temporal gyrus, but relatively intact white matter, suggesting early disturbances in cortical expansion underlying vulnerability to psychosis.^46^ Interestingly, Evermann et al. also showed that specific symptoms of PLEs (positive and depressive subscales) were associated with reductions in cortical gyrification in precuneus, right supramarginal, and temporal regions, but that cognition did not significantly mediate this relationship.^37^ While these studies advanced our understanding of PLEs, most are limited by their sample size and cross-sectional design. In addition, only a few have examined changes in cortical gyrification relating to PLEs in adolescents—a crucial period of neurodevelopment and for the emergence of psychiatric disorders.

The present findings showed that adolescents with elevated CAPE-3 scores showed lower gyrification in the banks of superior temporal sulcus, middle temporal gyrus, superior frontal gyrus, and precentral gyrus in the left hemisphere; that persisted from mid-adolescence to late adolescence thus reflecting *static gyrification* changes. This is in the context of very widespread changes in cortical *dynamic* gyrification in the entire CAPE-3 group over this period, most especially in prefrontal cortices, reflecting normal maturational processes between mid-adolescence to late-adolescence. Thus, this difference in the developmental trajectory of gyrification in the current study could reflect either a delay in this normal maturational increase, or a persistent reduction in gyrification. Lower gyrification in these areas among the higher scorers is consistent with a recent finding of lower gyrification in left temporal lobe in a cohort of young adults with persistent PLEs^16^ and lower cortical gyrification in schizophrenia.^47,48^ Disruption of left fronto-temporal connectivity has been associated with the genesis of positive psychotic symptoms, particularly hallucinations.^49–51^ Contemporary theories of hallucinatory experience suggest that deficits in sensory gating and language processing result in a “feedforward” aberrant information transmission leading to auditory hallucinations^52^; deficits in salience network deficits resulting in aberrant salience that could lead to both hallucinations^52^ and delusions.^53^ A recent cross-species study demonstrated that variation in cortical gyrification is related to underlying strength of white matter connections^54^; and changes in fronto-temporal white matter connectivity has been reported to be implicated in the etiology of psychotic symptoms.^55^ However, this effect was not evident in our replication study of patients with first-episode psychosis suggesting lower gyrification in these areas may reflect a marker of sub-clinical PLEs only.

We also observed a CAPE × TIME interaction where high CAPE-3 scorers showed an increase in gyrification in the right superior and inferior parietal lobules. The parietal changes over time may reflect the specific role of the parietal cortex in efficient multimodal integration, working memory, and self-monitoring.^56^ As the adolescents in our study did not have clinical intensity of symptoms as observed in the full-blown clinical illness in schizophrenia, but were experiencing PLEs, it is possible that these increases in gyrification in the inferior parietal lobule may reflect compensatory mechanisms supplementary to the more widely observed age-related maturational changes and hence changes in *dynamic gyrification*. This compensatory increase in gyrification may be secondary to lower fronto-temporal gyrification in high PLE adolescents that may reflect the consequent need for enhanced self-monitoring of self-generated material—a function of the left superior temporal gyrus that is often disrupted in psychotic patients.^49–51^ Indeed, this compensatory increase in gyrification is supported by data demonstrating higher parietal cortical gyrification in healthy adults was associated with better functional capacity^57^ while it was associated with persistent auditory hallucinations in schizophrenia.^58^ In addition, we observed higher gyrification in right parietal cortex in our independent sample of patients with first-episode psychosis compared to HC, thus replicating the changes of *dynamic gyrification*. This suggests a potential compensatory mechanism that spans across high-risk and clinical populations, supporting similar structural changes across a spectrum of psychotic experience. It is recognized that development during adolescence involves significant structural brain changes and cortical gyrification which may be influenced by underlying changes in gray matter volume, white matter integrity, brain volume, and surface area,^59–61^ referred to as *dynamic gyrification*. The changes in *dynamic gyrification* in parietal cortex which appears at late adolescence and also seen in those with first-episode psychosis may therefore reflect changes in shape and curvature related to cortical morphology/white matter pathology. It is known that persistent PLEs increase the risk of transition to psychosis,^7–10^ a finding that is replicated here (high CAPE-3 scorers were 5.6 times more likely to transition to psychosis than low CAPE-3 scorers). These abnormalities in developmental trajectory of gyrification may therefore underlie PLEs in adolescents and provide a structural marker for vulnerability to psychosis.

The exploratory stratification using the entire CAPE-42 replicated the higher gyrification in right superior parietal lobule but not the persistent reduction in gyrification as seen in the CAPE-3 high scorers. Of interest, although there was a strong correlation between CAPE-3 and CAPE-42 scores, and both were based on the same threshold on the positive subscale, our study indicates that stratification by CAPE-3 scores isolates a different subset of adolescents than when stratified by CAPE-42 scores. Indeed, there were no correlations between total CAPE-42 score and gyrification in the total sample and fewer adolescents that transitioned to psychosis were classified as experiencing high PLEs using the CAPE-42. This is not surprising because the CAPE 42 indexes a very broad range of experiences, with an increased number of nonspecific symptoms, while the CAPE-3 is focused on specific symptoms most proximal to clinical symptoms observed in psychotic illness. In our study, the high CAPE-3 scoring sample was not different on any confounding factors such as IQ, alcohol, or cannabis use. However, sub-clinical depressive symptoms (ADRS scores) were higher in the high versus low CAPE-3 scorers. Our data therefore support the value in stratifying adolescents according to CAPE-3 item scores and suggests that changes in gyrification are specific to these positive symptoms.

### Limitations

First, PLEs, depression, and substance-use measures were available only at follow-up (age 19 years). It is unclear if some adolescents experienced PLEs at baseline or whether the lower gyrification in fronto-temporal regions contributed to PLEs at follow-up. The high and low CAPE-3 groups were relatively well matched for all key variables except for depressive symptoms. Additionally, although the main findings in the study relating to the CAPE-3 cohort are confirmed using robust non-parametric permutation methods (TFCE), most of the results are reported at a liberal statistical threshold (*P* < .001 uncorrected). This limitation may be a result of small effect sizes in cortical gyrification studies, heterogeneity between adolescents, and CAPE scores that were only measured at age 19 years follow-up—this reflects the difficulties in studying PLEs. Moreover, our analyses investigating transition to psychosis were restricted to the adolescents that were followed up at age 23 years. Although the percentage drop-out was similar between high (40% decrease) and low (38% decrease) CAPE-3 scorers, the large drop-out introduces (unavoidable) bias to the sample as the reasons for drop-out are unclear and may reflect worsening PLEs or other mental health issues. Future studies aiming to replicate the current findings are therefore needed. Finally, due to careful harmonization procedures conducted in advance between scanning sites, scanning site was controlled for in analyses using a commonly used regression approach to remove mean differences between sites. However more sophisticated harmonization procedures could be considered in future.

## Conclusion

Our study demonstrates that adolescents with PLEs show increased rates of transition to psychosis accompanied by a neurodevelopmental brain structural abnormality of lower gyrification in fronto-temporal regions in the left hemisphere and increased gyrification in parietal regions in the right hemisphere. This was particularly in relation to positive PLEs such as hearing voices, bizarre experiences, and persecutory ideas, instead of negative PLEs such as blunted emotions. These neurodevelopmental abnormalities, especially in parietal cortex, are relevant to the clinical symptoms of psychosis and could offer a biomarker for identifying adolescents at high risk of transitioning to psychosis.

## Supplementary Material

sbac132_suppl_Supplementary_Material

## References

[1] Hinterbuchinger B , Mossaheb N. Psychotic-like experiences: a challenge in definition and assessment. Front Psychiatry. 2021;12:582392. 33854445 10.3389/fpsyt.2021.582392 PMC8039445

[2] Fusar-Poli P , Borgwardt S, Bechdolf A, et al. The psychosis high-risk state: a comprehensive state-of-the-art review. JAMA Psychiatry. 2013;70(1):107–120. 23165428 10.1001/jamapsychiatry.2013.269 PMC4356506

[3] Pain O , Dudbridge F, Cardno AG, et al. Genome-wide analysis of adolescent psychotic-like experiences shows genetic overlap with psychiatric disorders. Am J Med Genet. 2018;177(4):416–425. 29603866 10.1002/ajmg.b.32630 PMC6001485

[4] Mackie CJ , Castellanos-Ryan N, Conrod PJ. Developmental trajectories of psychotic-like experiences across adolescence: impact of victimization and substance use. Psychol Med. 2011;41(1):47–58. 20346196 10.1017/S0033291710000449

[5] Kelleher I , Connor D, Clarke MC, Devlin N, Harley M, Cannon M. Prevalence of psychotic symptoms in childhood and adolescence: a systematic review and meta-analysis of population-based studies. Psychol Med. 2012;42(9):1857–1863. 22225730 10.1017/S0033291711002960

[6] Kelleher I , Keeley H, Corcoran P, et al. Clinicopathological significance of psychotic experiences in non-psychotic young people: evidence from four population-based studies. Br J Psychiatry. 2012;201(1):26–32. 22500011 10.1192/bjp.bp.111.101543

[7] Poulton R , Caspi A, Moffitt TE, Cannon M, Murray R, Harrington H. Children’s self-reported psychotic symptoms and adult schizophreniform disorder: a 15-year longitudinal study. Arch Gen Psychiatry. 2000;57(11):1053–1058. 11074871 10.1001/archpsyc.57.11.1053

[8] Scott J , Martin G, Welham J, et al. Psychopathology during childhood and adolescence predicts delusional-like experiences in adults: a 21-year birth cohort study. Am J Psychiatry. 2009;166(5):567–574. 19339357 10.1176/appi.ajp.2008.08081182

[9] Welham J , Scott J, Williams G, et al. Emotional and behavioural antecedents of young adults who screen positive for non-affective psychosis: a 21-year birth cohort study. Psychol Med. 2009;39(4):625–634. 18606046 10.1017/S0033291708003760

[10] Kaymaz N , Drukker M, Lieb R, et al. Do subthreshold psychotic experiences predict clinical outcomes in unselected non-help-seeking population-based samples? A systematic review and meta-analysis, enriched with new results. Psychol Med. 2012;42(11):2239–2253. 22260930 10.1017/S0033291711002911

[11] DeRosse P , Ikuta T, Karlsgodt KH, et al. White matter abnormalities associated with subsyndromal psychotic-like symptoms predict later social competence in children and adolescents. Schizophr Bull. 2017;43(1):152–159. 27190281 10.1093/schbul/sbw062 PMC5216847

[12] Drakesmith M , Dutt A, Fonville L, et al. Mediation of developmental risk factors for psychosis by white matter microstructure in young adults with psychotic experiences. JAMA Psychiatry. 2016;73(4):396–406. 26886143 10.1001/jamapsychiatry.2015.3375

[13] Nesvag R , Reichborn-Kjennerud T, Gillespie NA, et al. Genetic and environmental contributions to the association between cannabis use and psychotic-like experiences in young adult twins. Schizophr Bull. 2017;43(3):644–653. 27431873 10.1093/schbul/sbw101 PMC5464089

[14] Armstrong E , Schleicher A, Omran H, Curtis M, Zilles K. The ontogeny of human gyrification. Cereb Cortex. 1995;5(1):56–63. 7719130 10.1093/cercor/5.1.56

[15] Zilles K , Armstrong E, Schleicher A, Kretschmann HJ. The human pattern of gyrification in the cerebral cortex. Anat Embryol (Berl). 1988;179(2):173–179. 3232854 10.1007/BF00304699

[16] Fonville L , Drakesmith M, Zammit S, Lewis G, Jones DK, David AS. MRI indices of cortical development in young people with psychotic experiences: influence of genetic risk and persistence of symptoms. Schizophr Bull. 2019;45(1):169–179. 29385604 10.1093/schbul/sbx195 PMC6293214

[17] de Wit S , Ziermans TB, Nieuwenhuis M, et al. Individual prediction of long-term outcome in adolescents at ultra-high risk for psychosis: applying machine learning techniques to brain imaging data. Hum Brain Mapp. 2017;38(2):704–714. 27699911 10.1002/hbm.23410 PMC6866746

[18] Damme KSF , Gupta T, Nusslock R, Bernard JA, Orr JM, Mittal VA. Cortical morphometry in the psychosis risk period: a comprehensive perspective of surface features. Biol Psychiatry Cogn Neurosci Neuroimaging. 2019;4(5):434–443. 31054647 10.1016/j.bpsc.2018.01.003 PMC6506173

[19] Harris JM , Whalley H, Yates S, Miller P, Johnstone EC, Lawrie SM. Abnormal cortical folding in high-risk individuals: a predictor of the development of schizophrenia? Biol Psychiatry. 2004;56(3):182–189. 15271587 10.1016/j.biopsych.2004.04.007

[20] Sasabayashi D , Takayanagi Y, Takahashi T, et al. Increased occipital gyrification and development of psychotic disorders in individuals with an at-risk mental state: a multicenter study. Biol Psychiatry. 2017;82(10):737–745. 28709499 10.1016/j.biopsych.2017.05.018

[21] Bakker G , Caan MW, Vingerhoets WA, et al. Cortical morphology differences in subjects at increased vulnerability for developing a psychotic disorder: a comparison between subjects with ultra-high risk and 22q11.2 deletion syndrome. PLoS One. 2016;11(11):e0159928. 27828960 10.1371/journal.pone.0159928 PMC5102447

[22] Padula MC , Schaer M, Armando M, et al. Cortical morphology development in patients with 22q11.2 deletion syndrome at ultra-high risk of psychosis. Psychol Med. 2018;48(14):2375–2383. 29338796 10.1017/S0033291717003920

[23] Matsuda Y , Ohi K. Cortical gyrification in schizophrenia: current perspectives. Neuropsychiatr Dis Treat. 2018;14:1861–1869. 30050300 10.2147/NDT.S145273 PMC6055839

[24] Sasabayashi D , Takahashi T, Takayanagi Y, Suzuki M. Anomalous brain gyrification patterns in major psychiatric disorders: a systematic review and transdiagnostic integration. Transl Psychiatry. 2021;11(1):176. 33731700 10.1038/s41398-021-01297-8 PMC7969935

[25] *Welcome to the IMAGENStudy* . https://imagen-europe.com/. Accessed January 1, 2018.

[26] Mossaheb N , Becker J, Schaefer MR, et al. The Community Assessment of Psychic Experience (CAPE) questionnaire as a screening-instrument in the detection of individuals at ultra-high risk for psychosis. Schizophr Res. 2012;141(2-3):210–214. 22986044 10.1016/j.schres.2012.08.008

[27] Konings M , Bak M, Hanssen M, van Os J, Krabbendam L. Validity and reliability of the CAPE: a self-report instrument for the measurement of psychotic experiences in the general population. Acta Psychiatr Scand. 2006;114(1):55–61. 16774662 10.1111/j.1600-0447.2005.00741.x

[28] Mark W , Toulopoulou T. Psychometric properties of “community assessment of psychic experiences”: review and meta-analyses. Schizophr Bull. 2016;42(1):34–44. 26150674 10.1093/schbul/sbv088 PMC4681550

[29] Yung AR , Nelson B, Baker K, Buckby JA, Baksheev G, Cosgrave EM. Psychotic-like experiences in a community sample of adolescents: implications for the continuum model of psychosis and prediction of schizophrenia. Aust N Z J Psychiatry. 2009;43(2):118–128. 19153919 10.1080/00048670802607188

[30] Wechsler D. Wechsler Intelligence Scale for Children. 4th ed. San Antonio, TX: Psychological Corporation; 2003.

[31] Saunders JB , Aasland OG, Babor TF, de la Fuente JR, Grant M. Development of the Alcohol Use Disorders Identification Test (AUDIT): WHO collaborative project on early detection of persons with harmful alcohol consumption--II. Addiction. 1993;88(6):791–804. 8329970 10.1111/j.1360-0443.1993.tb02093.x

[32] Bohn MJ , Babor TF, Kranzler HR. The Alcohol Use Disorders Identification Test (AUDIT): validation of a screening instrument for use in medical settings. J Stud Alcohol. 1995;56(4):423–432. 7674678 10.15288/jsa.1995.56.423

[33] Skinner HA. The drug abuse screening test. Addict Behav. 1982;7(4):363–371. 7183189 10.1016/0306-4603(82)90005-3

[34] Revah-Levy A , Birmaher B, Gasquet I, Falissard B. The Adolescent Depression Rating Scale (ADRS): a validation study. BMC Psychiatry. 2007;7(1):2. 17222346 10.1186/1471-244X-7-2 PMC1785378

[35] Schumann G , Loth E, Banaschewski T, et al. The IMAGEN study: reinforcement-related behaviour in normal brain function and psychopathology. Mol Psychiatry. 2010;15:1128–1139. 21102431 10.1038/mp.2010.4

[36] Luders E , Thompson PM, Narr KL, Toga AW, Jancke L, Gaser C. A curvature-based approach to estimate local gyrification on the cortical surface. Neuroimage. 2006;29(4):1224–1230. 16223589 10.1016/j.neuroimage.2005.08.049

[37] Evermann U , Gaser C, Besteher B, Langbein K, Nenadić I. Cortical gyrification, psychotic-like experiences, and cognitive performance in nonclinical subjects. Schizophr Bull. 2020;46(6):1524–1534. 32691058 10.1093/schbul/sbaa068 PMC7707080

[38] Desikan RS , Segonne F, Fischl B, et al. An automated labeling system for subdividing the human cerebral cortex on MRI scans into gyral based regions of interest. Neuroimage. 2006;31(3):968–980. 16530430 10.1016/j.neuroimage.2006.01.021

[39] IBM. *IBM SPSS Statistics for Macintosh*. Version 25.0 ed. Armonk, NY: IBM Corp.; 2017.

[40] Caverzasi E , Battistella G, Chu SA, et al. Gyrification abnormalities in presymptomatic c9orf72 expansion carriers. *J Neurol Neurosurg Psychiatry.* 2019;90(9):1005–1010. 31079065 10.1136/jnnp-2018-320265 PMC6820159

[41] Lee SE , Sias AC, Mandelli ML, et al. Network degeneration and dysfunction in presymptomatic C9ORF72 expansion carriers. Neuroimage Clin. 2017;14:286–297. 28337409 10.1016/j.nicl.2016.12.006 PMC5349617

[42] Smith SM , Nichols TE. Threshold-free cluster enhancement: addressing problems of smoothing, threshold dependence and localisation in cluster inference. Neuroimage. 2009;44(1):83–98. 18501637 10.1016/j.neuroimage.2008.03.061

[43] Thomas M , Szentgyorgyi T, Vanes LD, et al. Cognitive performance in early, treatment-resistant psychosis patients: could cognitive control play a role in persistent symptoms? Psychiatry Res. 2021;295:113607. 33285345 10.1016/j.psychres.2020.113607

[44] Sheehan DV , Lecrubier Y, Sheehan KH, et al. The Mini-International Neuropsychiatric Interview (M.I.N.I.): the development and validation of a structured diagnostic psychiatric interview for DSM-IV and ICD-10. J Clin Psychiatry. 1998;59:22–33;quiz 34. 9881538

[45] Andrade C. Understanding relative risk, odds ratio, and related terms: as simple as it can get. J Clin Psychiatry. 2015;76(7):e857–e861. 26231012 10.4088/JCP.15f10150

[46] Fonville L , Drakesmith M, Zammit S, Lewis G, Jones DK, David AS. MRI indices of cortical development in young people with psychotic experiences: influence of genetic risk and persistence of symptoms. Schizophr Bull. 2019;45(1):169–179. 29385604 10.1093/schbul/sbx195 PMC6293214

[47] White T , Hilgetag CC. Gyrification and neural connectivity in schizophrenia. Dev Psychopathol. 2011;23(1):339–352. 21262059 10.1017/S0954579410000842

[48] Cao B , Mwangi B, Passos IC, et al. Lifespan gyrification trajectories of human brain in healthy individuals and patients with major psychiatric disorders. Sci Rep. 2017;7(1):511. 28360420 10.1038/s41598-017-00582-1 PMC5428697

[49] Simons CJ , Tracy DK, Sanghera KK, et al. Functional magnetic resonance imaging of inner speech in schizophrenia. Biol Psychiatry. 2010;67(3):232–237. 19846064 10.1016/j.biopsych.2009.09.007

[50] Shergill SS , Brammer MJ, Fukuda R, Williams SC, Murray RM, McGuire PK. Engagement of brain areas implicated in processing inner speech in people with auditory hallucinations. Br J Psychiatry. 2003;182:525–531. 12777344 10.1192/bjp.182.6.525

[51] Shergill SS , Brammer MJ, Fukuda R, et al. Modulation of activity in temporal cortex during generation of inner speech. Hum Brain Mapp. 2002;16(4):219–227. 12112764 10.1002/hbm.10046 PMC6871832

[52] Tracy DK , Shergill SS. Mechanisms underlying auditory hallucinations-understanding perception without stimulus. Brain Sci. 2013;3(2):642–669. 24961419 10.3390/brainsci3020642 PMC4061847

[53] So SH , Chau AKC, їPeters ER, Swendsen J, Garety PA, Kapur S. Moment-to-moment associations between negative affect, aberrant salience, and paranoia. Cogn Neuropsychiatry. 2018;23(5):299–306. 30047842 10.1080/13546805.2018.1503080

[54] Van Essen DC , Donahue CJ, Coalson TS, Kennedy H, Hayashi T, Glasser MF. Cerebral cortical folding, parcellation, and connectivity in humans, nonhuman primates, and mice. Proc Natl Acad Sci USA. 2019;116(52):26173–26180. 31871175 10.1073/pnas.1902299116 PMC6936571

[55] Jones DK , Catani M, Pierpaoli C, et al. Age effects on diffusion tensor magnetic resonance imaging tractography measures of frontal cortex connections in schizophrenia. Hum Brain Mapp. 2006;27(3):230–238. 16082656 10.1002/hbm.20179 PMC6871456

[56] Ribolsi M , Lisi G, Di Lorenzo G, et al. Perceptual pseudoneglect in schizophrenia: candidate endophenotype and the role of the right parietal cortex. Schizophr Bull. 2013;39(3):601–607. 22419195 10.1093/schbul/sbs036 PMC3627750

[57] Green S , Blackmon K, Thesen T, et al. Parieto-frontal gyrification and working memory in healthy adults. Brain Imaging Behav. 2018;12(2):303–308. 28290070 10.1007/s11682-017-9696-9

[58] Kubera KM , Thomann PA, Hirjak D, et al. Cortical folding abnormalities in patients with schizophrenia who have persistent auditory verbal hallucinations. Eur Neuropsychopharmacol. 2018;28(2):297–306. 29305294 10.1016/j.euroneuro.2017.12.009

[59] Gautam P , Anstey KJ, Wen W, Sachdev PS, Cherbuin N. Cortical gyrification and its relationships with cortical volume, cortical thickness, and cognitive performance in healthy mid-life adults. Behav Brain Res. 2015;287:331–339. 25804360 10.1016/j.bbr.2015.03.018

[60] Schultz CC , Wagner G, Schachtzabel C, et al. Increased white matter radial diffusivity is associated with prefrontal cortical folding deficits in schizophrenia. Psychiatry Res Neuroimaging. 2017;261:91–95. 28171781 10.1016/j.pscychresns.2017.01.011

[61] Toro R , Perron M, Pike B, et al. Brain size and folding of the human cerebral cortex. Cereb Cortex. 2008;18(10):2352–2357. 18267953 10.1093/cercor/bhm261

