# Correction to: Psychotic Like Experiences in Healthy Adolescents are Underpinned by Lower Fronto-Temporal Cortical Gyrification: a Study from the IMAGEN Consortium

**DOI:** 10.1093/schbul/sbac189

**Published:** 2022-11-17

This is a correction to: Raka Maitra, Charlotte M Horne, Owen O’Daly, Evangelos Papanastasiou, Christian Gaser, IMAGEN list of authors, IMAGEN Consortium, Psychotic Like Experiences in Healthy Adolescents are Underpinned by Lower Fronto-Temporal Cortical Gyrification: a Study from the IMAGEN Consortium, *Schizophrenia Bulletin*, 2022;, sbac132, https://doi.org/10.1093/schbul/sbac132.

In the originally published version of this manuscript, author, Sukhi Shergill, was not properly acknowledged in the PDF and HTML version of the papers. This error has been corrected.

