# Supplementary material for: Psychotic Like Experiences in Healthy Adolescents are Underpinned by Lower Fronto-Temporal Cortical Gyrification: a Study from the IMAGEN Consortium
Source: Schizophr Bull. 2022 Oct 5;49(2):309–18. doi: 10.1093/schbul/sbac132 (PMC10016412; doi:10.1093/schbul/sbac132)
Supplement: sbac132_suppl_Supplementary_Material [file sbac132_suppl_supplementary_material.docx]

Supplement

Appendix: Gyrification in individuals at high risk for psychosis

| Author | N | Age | Determination of high risk | Sample | Method | Gyrification | Regions |
| --- | --- | --- | --- | --- | --- | --- | --- |
| Harris et al (2004)(1) | 30 | 16-25 yrs. | Relatives of patients with schizophrenia | (14) High risk vs (16) Transition to psychosis | Manual tracing | Increased gyrification in transition to psychosis | Right Prefrontal cortex |
| Bakker et al (2016)(2) | 60 | 19-28 yrs. | Structured Interview for Prodromal Syndromes (SIPS) | (18)22q11DS vs (18)UHR vs (24)HC | Automated, Freesurfer 5.3 | No difference in gyrification in UHR vs HC | - |
| Padula et al (2018)(3) | 44 | 11-21yrs. | Structured Interview for Prodromal Syndromes (SIPS) | (22)22q1DS-UHR vs (22)22q1DS-nonUHR | Automated, Freesurfer (version unspecified) | No difference in gyrification in UHR vs non UHR | - |
| Sasabayashi et al (2017)(4) | 208 | 16-27 yrs. | Comprehensive Assessment of At-Risk Mental States (CAARMS) | 83 ARMS vs 21 Transition to psychosis vs 104 HC | Automated, Freesurfer 5.3 | Increased gyrification in ARMS vs HC ;  Gyrification in occipital region (ARMS vs Transition to psychosis) | Bilateral frontal, temporal, parietal, and occipital regions |
| De Wit et al (2017) (5) | 126 | 12-18 yrs. | Structured Interview for Prodromal Syndromes (SIPS),  Family Interview for Genetic Studies and Bonn Scale for the Assessment of Basic Symptoms‐Prediction List;  Global assessment of functioning (GAF) | (64)UHR vs (62)HC; At 6 yrs. follow up- (17) Resilient UHR vs (24)Non resilient UHR | Automated and manual edits,  Freesurfer 5.1.0 ; machine learning | Reduced gyrification and increased gyrification in different areas between resilient and non-resilient UHR ; Gyrification shows 69% sensitivity, 78% specificity, 73% accuracy (p<0.01) in differentiating resilient and non-resilient UHR and predicting 6 yr. outcome | Widespread changes |
| Damme et al (2019)(6) | 81 | 18-20 yrs. | Structured Interview for Prodromal Syndromes (SIPS) | (43) High risk vs (38) HC | Automated, Freesurfer 6.0 | Reduced gyrification | Lateral orbitofrontal cortex, Bank of the superior temporal sulcus, Anterior isthmus of the cingulate gyrus, and temporal poles |
| Das et al (2018)(7) | 161 | 20-32 yrs. | Basel Screening Instrument for Psychosis (BSIP) | (63) High risk vs (16) transition to psychosis and (38) drug naïve FEP and (44)HC | Automated, Freesurfer 5.3 | No difference in gyrification | - |
| Fonville et al (2018) (8) | 247 | 18-20 yrs. | Psychosis-Like Symptom Interview (PLiKSi) | (136) PLE vs (111) HC | Automated, Freesurfer 5.3 | Reduced gyrification | Left temporal lobe. |
| Evermann et al,(2020)(9) | 103 | 20-40 yrs. | Community assessment of Psychic experiences (CAPE) | 103 HC | Automated, CAT12 | Reduced gyrification | Left precuneus, Right supramarginal and temporal area. |

UHR: Ultra high risk, ARMS: At risk mental state, HC: healthy controls, FEP: First episode psychosis

**Methods:**

**Stratification of samples on the basis of CAPE scores**

First, we stratified our sample using the upper and lower quartiles of the composite scores of items 5, 7 and 33 from the CAPE questionnaire (hereon referred to as the CAPE-3 sample). This was based on a previous study that performed a factor analysis on the full CAPE questionnaire and showed that three factors; “Bizarre Experiences”, “Perceptual Abnormalities”, and “Persecutory Ideas” were most specific for predicting poor outcomes in adolescents experiencing PLEs (10). A recent meta-analysis then reported that these three factors could be reliably captured by three items on the CAPE positive subscale relating to “Messages”, “Persecutory ideations” and “Hearing voices” (items 5, 7 and 33) (11). It has been reported that a score of 2 on each item of the CAPE positive subscale, combining frequency and distress, provides adequate positive predictive value for transition to psychosis (12). As CAPE scores are skewed, samples are often stratified as upper and lower quartiles with the upper quartile (high scorers) showing high psychotic proneness and lower quartile (low scorers) showing low psychotic proneness (13). Therefore, we adopted this approach and stratified participants into high and low scorers based on the upper and lower quartiles of the CAPE-3 scale. High scorers had scores ranging between 3 ‐ 16, corresponding to an itemized score range of 1 ‐ 5.33 (14).

Second, we stratified the sample according to the full CAPE scale (hereafter referred to as the CAPE-42 sample). We carried out an exploratory analysis with the high and low scores based on the upper and lower deciles of the total CAPE score; similar to analyses used in previous studies (14, 15).

**Measurement of cortical gyrification**

CAT12 pipeline extracts the central surface(16) implementing further topology correction(17) and spherical mapping(18). Local gyrification was calculated as absolute mean curvature (19) at each vertex across the central surface mesh model. Mean curvature at a given vertex is defined as:


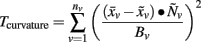


where 
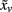
 is the centroid of its neighbors of vertex v, B_v_is the average distance from the centroid of each of the neighbors, and “·” is the vector product operator(20, 21)

**Additional exploratory analyses**

1. **High vs Low CAPE 42 scorers**

Exploratory analyses were performed with high and low scorers of CAPE-42. This was performed using a similar flexible factorial model in SPM but using CAPE-42 10^th^ decile score grouping as the between-subject factor (high and low scorers). IQ (WISC scores) was added as a covariate of no interest to this model as IQ was significantly different between high and low CAPE-42 scorers (but not between CAPE-3 groups). Finally, a separate exploratory analysis of the parametric effect of PLEs on cortical gyrification was performed using the entire sample of 1252 adolescents (i.e., using PLEs as a continuous variable).

Results

For the exploratory analyses using CAPE-42 sample stratification, there was a total sample of 247 adolescents with 123 high CAPE scorers and 124 low scorers. There were significant differences between high and low scorers for depressive symptoms, alcohol use, drug use and IQ. Thus, stratification by CAPE-3 provides the better matched sample for the analyses. In the entire sample of 1252 adolescents, CAPE-42 scores showed a moderate negative correlation with depressive symptom scores and moderate positive correlations with alcohol, cannabis use and IQ.

*Exploratory analyses based on CAPE-42 scorers*

A main effect of group (F (1,245)= 10.96, p<0.001 uncorrected) was observed in left precentral and right superior parietal areas. There was a significant main effect of time (FWE at p<0.05) in the same areas as the earlier CAPE-3 analysis results (see paper), including prefrontal regions. There was also a group X time interaction (F (1,245) = 11.09, p<0.001 uncorrected) in medial orbitofrontal in the right hemisphere. Post hoc analysis showed high scorers had higher gyrification in left precentral and right superior parietal cortex at both baseline and follow up visits (t(1,245) =3.11, p<0.001 uncorrected).

1. **Whole sample analysis**

In the entire sample of 1252 adolescents, a paired t-test between baseline and follow up replicated the main effect of TIME in CAPE-3 analysis (t(1,1251) = 4.11, FWE corrected p < 0.05). There was no significant correlation between changes in cortical gyrification and the normalized total CAPE-42 scores.

1. **Independent sample study**

Participants

Participant exclusion criteria included a history of neurological illness, diagnosed drug dependency within the past six months, current major physical illness and contraindication for MRI. The exclusion criteria for the HC also included a history of psychiatric illness or having a first-degree relative that currently or previously suffered from a psychotic illness. Patients were recruited from the South London and Maudsley (SLaM) NHS foundation trust, Oxleas NHS Foundation Trust and North East London NHS Foundation Trust (NELFT). Ethical approval was obtained by the London Camberwell St. Giles Research Ethics Committee and all participants provided written informed consent prior to participation. Demographic information for this sample is reported elsewhere (22).

Methods

Cortical gyrification was measured as described above. Statistical analysis was also performed using General linear modelling in SPM where an independent sample t-test was used to compare cortical gyrification between psychosis patient and HC groups; with age added as a covariate of no interest to the model.

**The importance of CAPE 3 items stratification**

The exploratory stratification using the entire CAPE-42 replicated the higher gyrification in right superior parietal lobule but not the persistent reduction in gyrification as seen in the CAPE-3 high scorers. Although there was a strong correlation between CAPE-3 and CAPE-42 scores, and both were based on the same threshold on the positive subscale, our study indicates that stratification by CAPE-3 scores isolates a different subset of adolescents than when stratified by CAPE-42 scores. Indeed, there were no correlations between total CAPE-42 score and gyrification in the total sample and fewer adolescents that transitioned to psychosis were classified as experiencing high PLEs using the CAPE-42. This is not surprising because the CAPE 42 indexes a very broad range of experiences, with an increased number of non-specific symptoms, while the CAPE-3 is focused on specific symptoms most proximal to clinical symptoms observed in psychotic illness. In our study, the high CAPE-3 scoring sample was not different on any confounding factors such as IQ, alcohol or cannabis use. However, sub-clinical depressive symptoms (ADRS scores) were higher in the high versus low CAPE-3 scorers. Our data therefore support the value in stratifying adolescents according to CAPE-3 item scores and suggests that changes in gyrification are specific to these positive symptoms.

References:

1. Harris JM, Whalley H, Yates S, Miller P, Johnstone EC, Lawrie SM. Abnormal cortical folding in high-risk individuals: a predictor of the development of schizophrenia? Biol Psychiatry. 2004;56(3):182-9.

2. Bakker G, Caan MW, Vingerhoets WA, da Silva-Alves F, de Koning M, Boot E, et al. Cortical Morphology Differences in Subjects at Increased Vulnerability for Developing a Psychotic Disorder: A Comparison between Subjects with Ultra-High Risk and 22q11.2 Deletion Syndrome. PLoS One. 2016;11(11):e0159928.

3. Padula MC, Schaer M, Armando M, Sandini C, Zoller D, Scariati E, et al. Cortical morphology development in patients with 22q11.2 deletion syndrome at ultra-high risk of psychosis. Psychol Med. 2018;48(14):2375-83.

4. Sasabayashi D, Takayanagi Y, Takahashi T, Koike S, Yamasue H, Katagiri N, et al. Increased Occipital Gyrification and Development of Psychotic Disorders in Individuals With an At-Risk Mental State: A Multicenter Study. Biol Psychiatry. 2017;82(10):737-45.

5. de Wit S, Ziermans TB, Nieuwenhuis M, Schothorst PF, van Engeland H, Kahn RS, et al. Individual prediction of long-term outcome in adolescents at ultra-high risk for psychosis: Applying machine learning techniques to brain imaging data. Hum Brain Mapp. 2017;38(2):704-14.

6. Damme KSF, Gupta T, Nusslock R, Bernard JA, Orr JM, Mittal VA. Cortical Morphometry in the Psychosis Risk Period: A Comprehensive Perspective of Surface Features. Biol Psychiatry Cogn Neurosci Neuroimaging. 2019;4(5):434-43.

7. Das T, Borgwardt S, Hauke DJ, Harrisberger F, Lang UE, Riecher-Rossler A, et al. Disorganized Gyrification Network Properties During the Transition to Psychosis. JAMA Psychiatry. 2018;75(6):613-22.

8. Fonville L, Drakesmith M, Zammit S, Lewis G, Jones DK, David AS. MRI Indices of Cortical Development in Young People With Psychotic Experiences: Influence of Genetic Risk and Persistence of Symptoms. Schizophr Bull. 2018.

9. Evermann U, Gaser C, Besteher B, Langbein K, Nenadić I. Cortical Gyrification, Psychotic-Like Experiences, and Cognitive Performance in Nonclinical Subjects. Schizophr Bull. 2020.

10. Yung AR, Nelson B, Baker K, Buckby JA, Baksheev G, Cosgrave EM. Psychotic-like experiences in a community sample of adolescents: implications for the continuum model of psychosis and prediction of schizophrenia. Aust N Z J Psychiatry. 2009;43(2):118-28.

11. Mark W, Toulopoulou T. Psychometric Properties of "Community Assessment of Psychic Experiences": Review and Meta-analyses. Schizophr Bull. 2016;42(1):34-44.

12. Mossaheb N, Becker J, Schaefer MR, Klier CM, Schloegelhofer M, Papageorgiou K, et al. The Community Assessment of Psychic Experience (CAPE) questionnaire as a screening-instrument in the detection of individuals at ultra-high risk for psychosis. Schizophrenia Research. 2012;141(2-3):210-4.

13. Modinos G, Ormel J, Aleman A. Altered activation and functional connectivity of neural systems supporting cognitive control of emotion in psychosis proneness. Schizophr Res. 2010;118(1-3):88-97.

14. Papanastasiou E, Mouchlianitis E, Joyce DW, et al. Examination of the neural basis of psychoticlike experiences in adolescence during reward processing. JAMA Psychiatry. 2018.

15. Papanastasiou E, Mouchlianitis E, Joyce DW, McGuire P, Boussebaa C, Banaschewski T, et al. Examination of the neural basis of psychotic-like experiences in adolescence during processing of emotional faces. Scientific Reports. 2020;10(1):5164.

16. Dahnke R, Yotter RA, Gaser C. Cortical thickness and central surface estimation. Neuroimage. 2013;65:336-48.

17. Yotter RA, Dahnke R, Thompson PM, Gaser C. Topological correction of brain surface meshes using spherical harmonics. Human brain mapping. 2011;32(7):1109-24.

18. Yotter RA, Thompson PM, Gaser C. Algorithms to improve the reparameterization of spherical mappings of brain surface meshes. J Neuroimaging. 2011;21(2):e134-47.

19. Do Carmo MP. Differential Geometry of Curves and Surfaces. Prentice Hall, NJ: Englewood Cliffs.; 1976.

20. Luders E, Kurth F, Mayer E, Toga A, Narr K, Gaser C. The Unique Brain Anatomy of Meditation Practitioners: Alterations in Cortical Gyrification. Front Hum Neurosci. 2012;6(34).

21. Luders E, Thompson PM, Narr KL, Toga AW, Jancke L, Gaser C. A curvature-based approach to estimate local gyrification on the cortical surface. Neuroimage. 2006;29(4):1224-30.

22. Thomas M, Szentgyorgyi T, Vanes LD, Mouchlianitis E, Barry EF, Patel K, et al. Cognitive performance in early, treatment-resistant psychosis patients: Could cognitive control play a role in persistent symptoms? Psychiatry Res. 2021;295:113607.
